# Landslide detection and inventory updating using the time-series InSAR approach along the Karakoram Highway, Northern Pakistan

**DOI:** 10.1038/s41598-023-34030-0

**Published:** 2023-05-09

**Authors:** Sajid Hussain, Bin Pan, Zeeshan Afzal, Muhammad Ali, Xianlong Zhang, Xianjian Shi, Muhammad Ali

**Affiliations:** 1 School of Remote Sensing and Information Engineering, Wuhan University, Wuhan, 430079 China; 2 State Key Laboratory of Information Engineering in Surveying, Mapping and Remote Sensing, Wuhan University, Wuhan, 430079 China; 3 Dipartimento di Ingegneria, Università degli Studi di Napoli Parthenope, 80133 Naples, Italy; 4 Department of Geology and Geography, West Virginia University, Morgantown, WV 26505 USA

**Keywords:** Climate sciences, Natural hazards, Environmental impact

## Abstract

Karakoram Highway (KKH) is frequently disrupted by geological hazards mainly landslides which pose a serious threat to its normal operation. Using documented inventory, optical imagery interpretation, and frequency-area statistics, the features of slope failure, the spatial distribution, and their link to numerous contributing factors have all been effectively explored along the KKH. An updated inventory for the area was recreated using the interferometric synthetic aperture radar (InSAR) persistent scatterer (PS) technology to further investigate millimetre-accurate measurements of slope deformation (V_slope_). Utilizing the PS approach, Sentinel-1 data from Jan 2018 to Jan 2022 were processed by which we obtained a deformation rate (V_Slope_) that varies between 0 and 364 mm/year. A total number of 234 landslides were cited from the literature and classified while 29 new potential landslides were detected and several pre-existing landslides were redefined by the InSAR approach, which was incorporated to generate an updated landslide susceptibility model with 86.6% of prediction precision in the area under curve method. As previous studies done by applying the InSAR technique incorporated a short span temporally and they missed some highly deforming zones like Budalas and Khanabad landslides, contain mean velocities > 50 mm/yr, which we studied individually in this work. In this study, a comprehensive application of the InSAR technique to assessing its performance in detecting and analysing landslides has been applied. The deformation velocity (V_slope_) model shows high displacement in some regions, which needed to be further investigated by geoscientists, and the updated developed landslide inventory and susceptibility map can be used for land use planning and landslide mitigation strategies.

## Introduction

Landslides are the major natural disaster of the world caused by gravitational force as well as other factors like precipitation, earthquakes, or human activities. According to official statistics, landslides have resulted in more than 18,000 fatalities globally between 1998 and 2017^1^, and worldwide insured property damages from hydrological disasters, including landslides, exceed $127 billion since 1980^2^. The “One Belt and One Road” initiative’s “China–Pakistan Economic Corridor” (CPEC) serves as its centerpiece project, connecting China and Pakistan via the Karakoram Highway (KKH). However, on this important road, the high mountain topography, a lot of loose debris, and sudden, intense rainfall are causing frequent and catastrophic geological disasters such as rock collapses, glacier debris flows, landslides, debris creep, soil creep, and, in rare circumstances, avalanches^3^. Very huge rockslides or rock falls have caused over 115 rock avalanches since 1987^4^, the KKH experienced considerable damage due to earthquake-induced landslides in 2005^5^, and a huge landslide in Attabad in 2010 blocked the river and formed a barrier lake that was more than 20 km long, flooding the roadway and impeding traffic^6^. Roads, settlements, and the surrounding environment sustain significant damage as a result of all of these disasters.

Landslide inventory maps for the area have been developed by many researchers by applying different methods^4^. designed a conventional geomorphological method to map the landslide^7–10^, incorporated optical remote sensing interpretation to generate landslide inventory for the area. The precision and extent of landslide mapping using conventional remote sensing techniques are, however, limited for some reasons, including the lack of discernible spectral signatures, the presence of various types of land cover, the chances of missing inventory data, and the influence of meteorological conditions. Due to an incomplete inventory of landslides and the high level of uncertainty in optical remote sensing interpretations, determining landslide susceptibility is difficult. The creation of landslide susceptibility and geohazard maps has been impeded in many parts of the world by incomplete landslide inventories^11^. For engineers and geologists, determining landslide susceptibility with limited data is a problem in the support of planners and governmental organizations. To undertake landslide detection and mapping, optical remote sensing can be integrated with InSAR technology, which can overcome these limitations^12^. A strong tool for landslide detection and mapping at a wide scale is the use of SAR interferometric techniques, which can also help with the development and updating of landslide inventory maps^13^.

The identification and monitoring of slope deformation are essential for reducing the damages caused by landslides. However, it is quite difficult to identify and keep track of the frequently gradual deformation of many slopes. Landslides with significant morphological characteristics and deformation signs, such as armchair shapes, scarps, and surface cracks, can be visually interpreted and identified using optical remote sensing images, but it is challenging to assess whether the landslide is deforming or not and to calculate the deformation velocity^14^. Interferometric Synthetic Aperture Radar (InSAR) techniques have gained widespread acceptance and use as tools for landslide mapping and monitoring over recent years. Although different techniques of InSAR have been successfully used in mapping slope deformations associated like^14^ estimated surface deformation areas detected by SBAS-InSAR^12,15^, applied D-InSAR method for land subsidence and landslide monitoring^16–18^, generated SqeeInSAR method to check the ground movement^19^, used Stanford Method for Persistent Scatterers (StaMPS) to assess the deformation in dense vegetation area^20–22^, tested PSInSAR approach to calculate the movement of landslide. PSInSAR is effective in automatic slow-moving landslide mapping based on the use of a spatial statistical approach, the identification of individual landslides and the delineation of generalized unstable areas, the redefinition of the limits of ancient landslides, the identification of landslides based on the multi-temporal comparison of SAR imagery and the identification of the terrain elements responsible for slope deformation is made easier by the combined use of optical imagery^23^. Various forms of PSInSAR-related studies have been conducted to determine the spatial or temporal landslide deformation patterns or the kinematic resolution of slow-moving landslides to estimate the scale of these landslides^24^. The PSInSAR method is used because it has several benefits over other approaches for overcoming decorrelation issues and producing time series of phase changes independent of residual atmospheric and DEM (Digital Elevation Model) impacts^25^.

In this study, optical remote sensing interpretation and the PSInSAR approach were formulated to detect the landslides and develop an updated landslide inventory. The primary objectives were to map all kinds of landslides along the KKH (Gilgit to Khunjerab section) and to estimate displacement maps (V_slope_) that can be used to locate new landslides, unstable areas in general, and to redraw the boundaries of landslides that have already been located previously according to deformation model. The updated inventory was applied to assess the susceptibility mapping from very highly susceptible to low-susceptible zones. In order to comprehend the observed subsidence and its relationship to environmental and anthropogenic factors, the most responsible causative factors in all previous studies have been selected and analyzed. The final InSAR approach-based inventory will be helpful to track the particularly unstable bodies to mitigate disasters in the future.

## Study area

**Figure 1:**
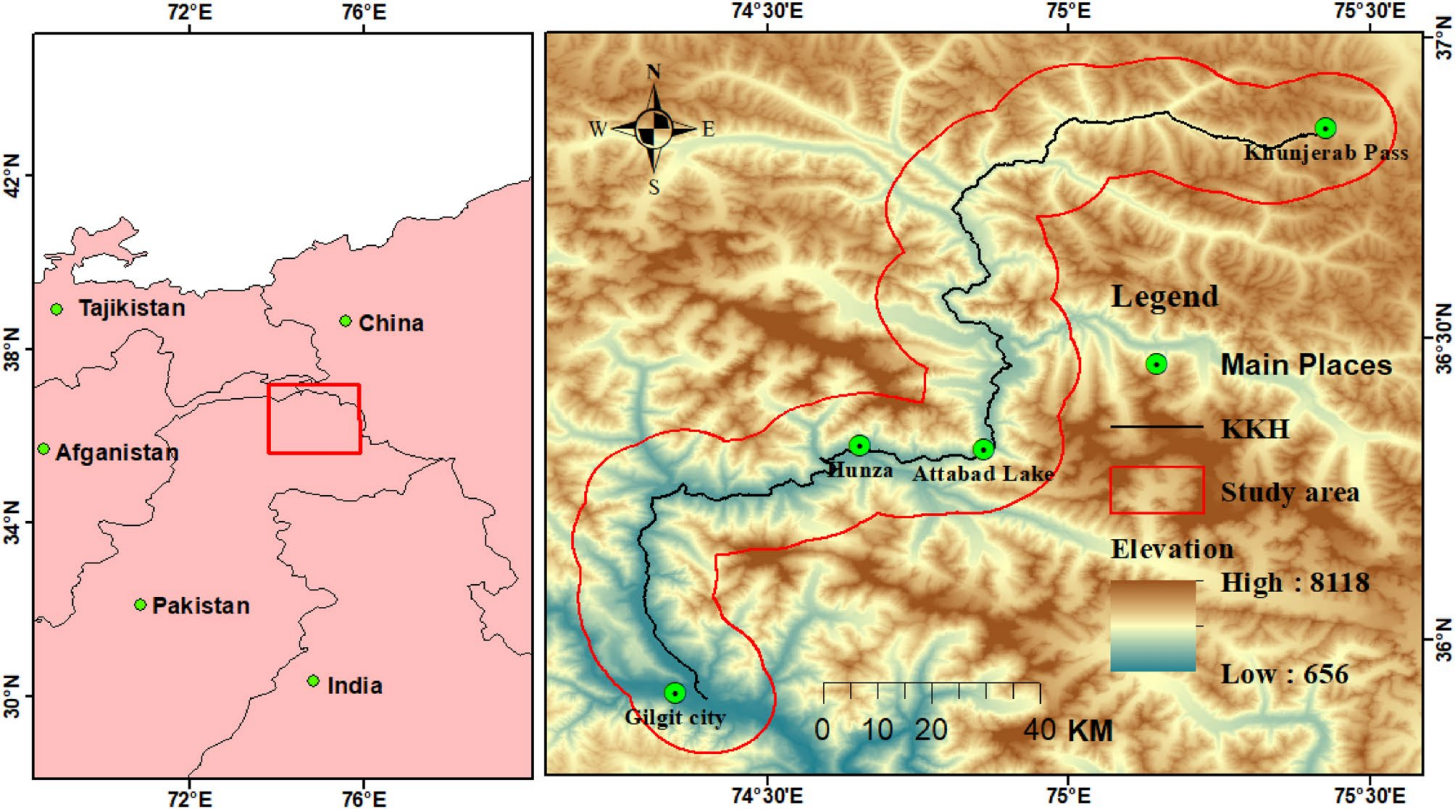
Location map of the study area portraying 10 km buffer zone (red solid polygon) along the KKH (Gilgit city to Khunjerab section). Solid red circle shows the main landslide areas while black solid line is Karakoram Highway. (Open Accessed SRTM DEM 30 m). (©USGS).

KKH in northern Pakistan is a significant part of the CPEC, it is frequently disrupted by numerous geological and hydro-climatological hazards. In this study, 10 km buffer of 263 km section of KKH from Gilgit city to Khunjerab pass was examined with an area of 4629 km^2^ (Fig. 1).

The region is situated in the active zone of collision between the Indian and Asian plates. With convergence and uplift rates of about 4–5 cm/year and about 7 mm/year respectively, crustal shortening, active faulting, and subduction are still occurring^26,27^. The Main Karakoram Thrust (MKT), Main Mantle Thrust (MMT), and Main Boundary Thrust (MBT) are the three main thrust fault belts, which together with river downcutting, are the primary forces determining the development and evolution of landforms in the area like glacial, periglacial, and fluvial landforms. The MKT and Karakorum Fault (KF) cause brittle deformation, which are the major tectonic features in this region, due to which rock masses are severely fractured and jointed^28^.

**Figure 2:**
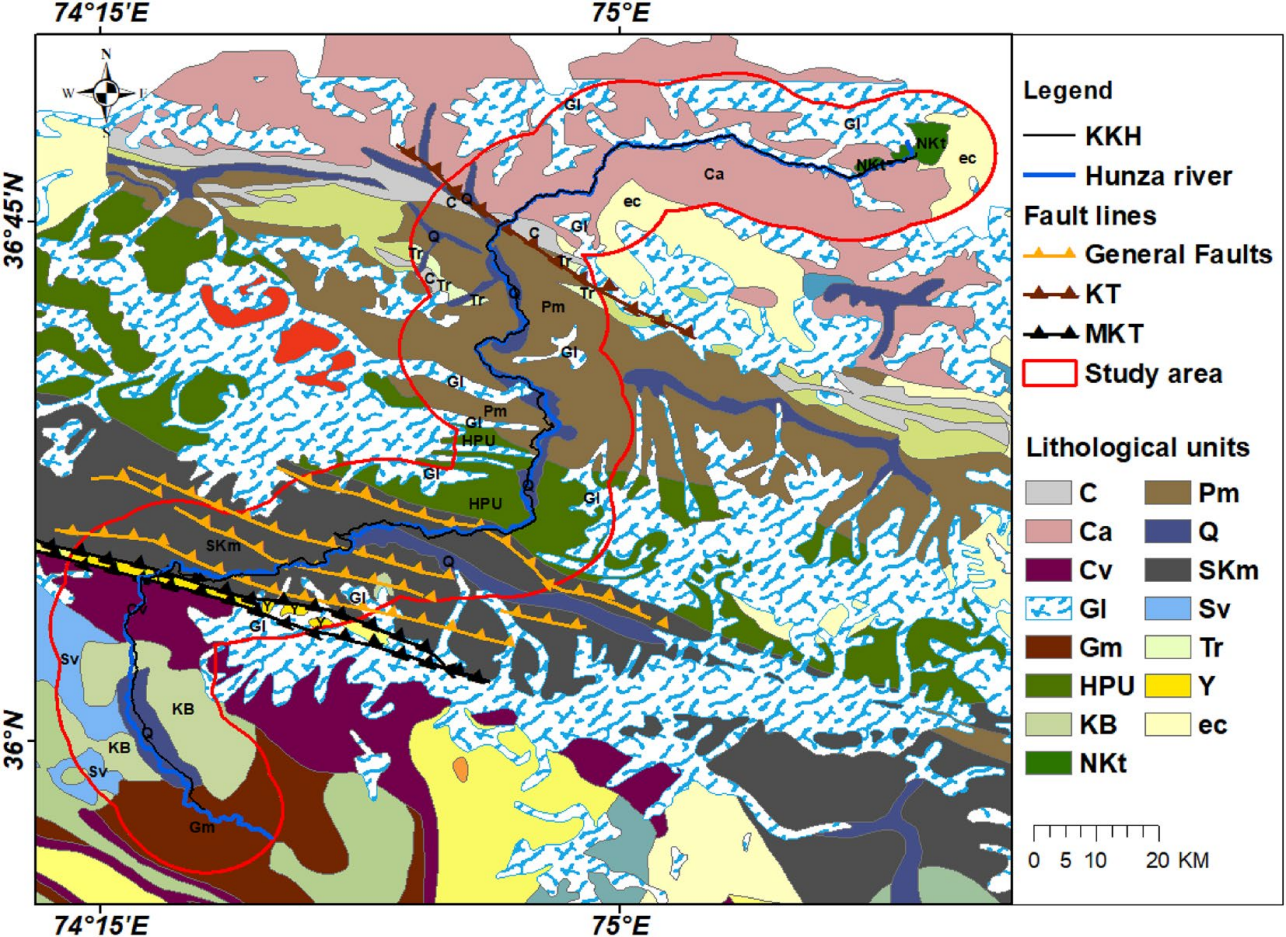
Regional Geological map of the area which shows the Hunza river, fault lines (KT is Karakoram Thrust and MKT is Main Karakoram Thrust), and lithological units in the study area, where C is Cretaceous Sandstones, Shales, and limestones, Ca is Devonian rocks, Cv is Chalt group, Gl represents Glaciers, Gm is Gilgit complex metasedimentary rocks, HPU stands for Hunza plutonic unit, KB is Kohistan Batholiths, NKt represents Northern Karakoram Terrane, Pm is Permian massive Limestone, Q stands for Quaternary deposits, SKm is Southern Karakoram complex, Sv shows Kohistan Arc sequence, Tr stands for Triassic massive limestone and dolomite, Y is Yasin group and ec represents Besal eclogites. (©Survey of Pakistan).

Lithology plays an important role in triggering landslides. The area is characterized by fractured and weathered rock masses possessing diverse igneous, metamorphic, and sedimentary rocks (Fig. 2). The Baltit Group, Chalt schists, Quaternary sediments, Gujhal dolomite, kilk formation, and deformed Misgar slates are the most prominent local lithologies, and they are all tectonically affected and responsible for slope destabilization alongside the highway^7^. The Southern Karakoram metamorphic Complex (SKm), the Hunza Plutonic Unit (HPU), the Shaksgam Formation (SF), and Quaternary (Q) Deposits comprise the region's geology^26^. Paragneises with interbedded pelites and amphibolite constitute the SKm. Permian massive limestones are part of the Shaksgam Formation, a region of the northern Karakoram landscape. Plagioclase, quartz, biotite, and hornblende are found in the HPU, a portion of the Karakoram batholith. Lithologies of different ages are exposed along the KKH have been weathered and weaken by seismic, hydro-climatological, and anthropogenic activities leads to huge landslides and land deformation in the area.

## Methods and materials

### Datasets

**Figure 3:**
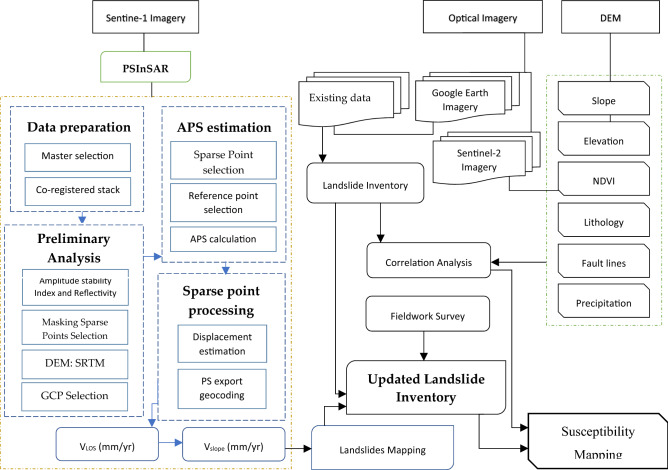
Methodology flow chart is applied in the study.

In this study, 4 years (Jan. 2018 to Jan. 2022) imagery of the C-band Sentinel-1 SAR dataset was downloaded from Alaska Satellite Facility (ASF) online system (https://search.asf.alaska.edu), which included scenes in ascending and descending paths as shown in Table 1. For the processing of SAR data for the ground deformation investigation and the InSAR time-series analysis, MATLAB and SARPROZ platforms were employed. The Shuttle Radar Topography Mission (SRTM) Digital Elevation Model (DEM) of the 30-m resolution was taken from the USGS database (https://earthexplorer.usgs.gov) to extract the topographic parameters in ArcGIS environment. To create landslide inventory Google Earth imagery was incorporated with the existing data which was validated during fieldwork. Sentinel-2 optical sensor data of 10-m resolution was also received from the USGS Earth Explorer database system for supervised landcover mapping in R-studio package. Rainfall and landslide events are directly proportion-related, so to evaluate it annual precipitation data was accessed via the Climate Hazards Group Infrared Precipitation with Station data (CHIRPS) online database system (https://www.chc.ucsb.edu/data/chirps). Geology maps and fault lines were extracted by the modification of maps designed by^12,26^ in ArcMAP 10.5 software. The methodological flowchart has shown in Fig. 3.

**Table 1:** Data used in the study.

Source	Sentinel-1A	Sentinel-1A	Google Earth	Sentinel-2A	DEM
Purpose	Time-series displacement assessment	Spatial pattern analysis of ground movement	Inventory development & Evolution precession	Landcover mapping & InSAR results visualization	Topographic parameters extraction
Flight direction	Descending	Ascending	–	Descending	–
Temporal span	1st Jan. 2018–23rd Dec. 2021	12th Jan. 2018–31st July 2021	2017–2022	13th Aug. 2021	11th Feb. 2000
Band	C	C	Visible	Visible	X
Wavelength (m)	0.056	0.056	–	0.443–2.19 (μm)	0.031
Spatial Resolution (m)	5*20	5*20	< 2	10	30
NO. of Images	110	108	–	1	–
Revisiting time (days)	12	12	–	2–5	11 (mission length)
Path	107	100	–	–	–
Frame	473	114	–	–	–
Incident angle (.)	39.64	44.16	–	–	57
Product type	SLC	SLC	–	–	–
Polarization	VV	VV	–	–	VV
Acq. mood	IW	IW	–	–	–
NO. of PS points	213,601	298,827	–	–	–
Min. LOS (mm/yr)	− 346	− 364	–	–	–
Max. LOS (mm/yr)	376	364	–	–	–

### Landslide inventory

**Figure 4:**
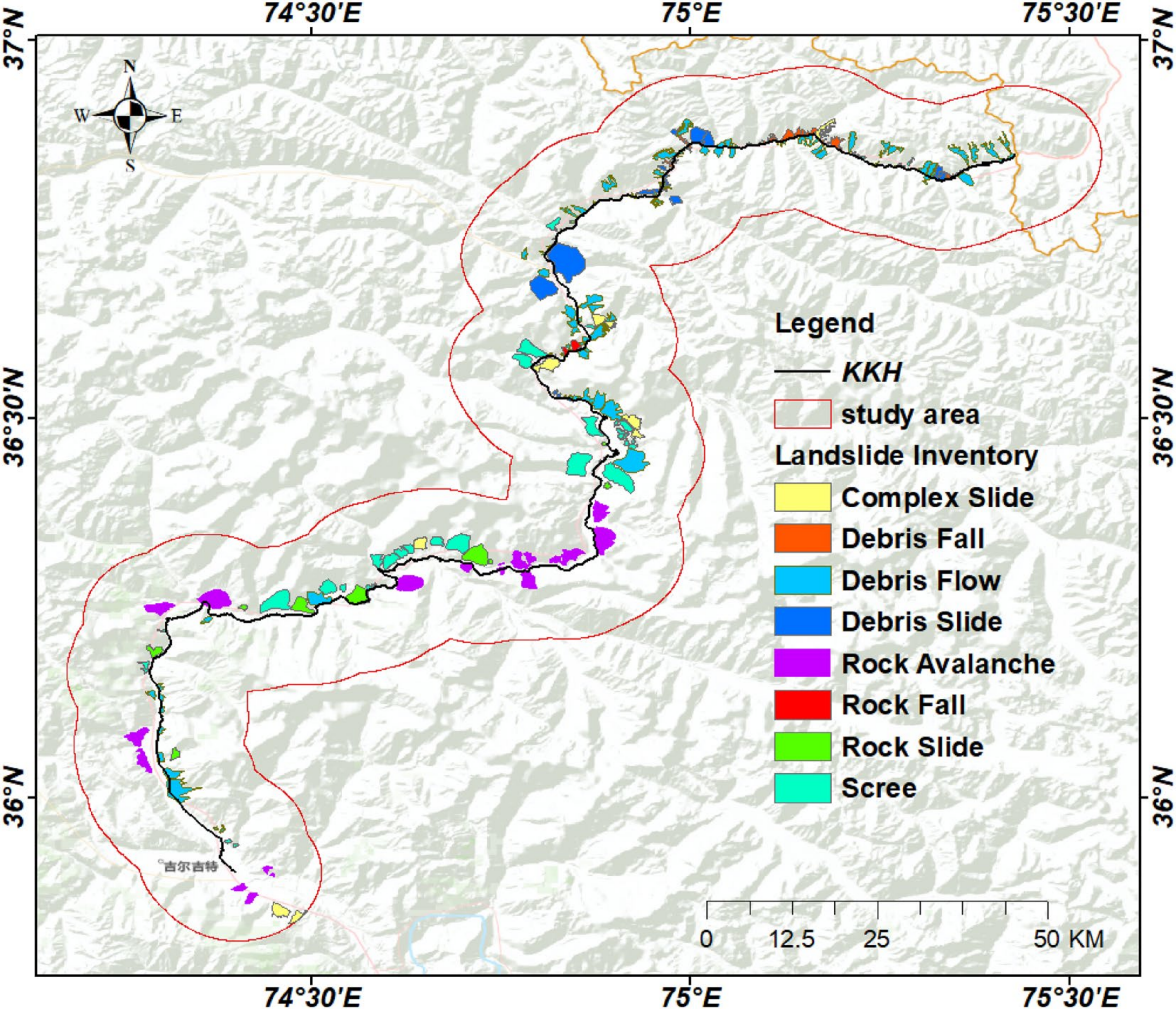
Landslide inventory classified based on movement along Karakoram Highway (black solid line) with different colors. Study area portraying 10 km buffer zone (red solid polygon) along the KKH.

**Figure 5:**
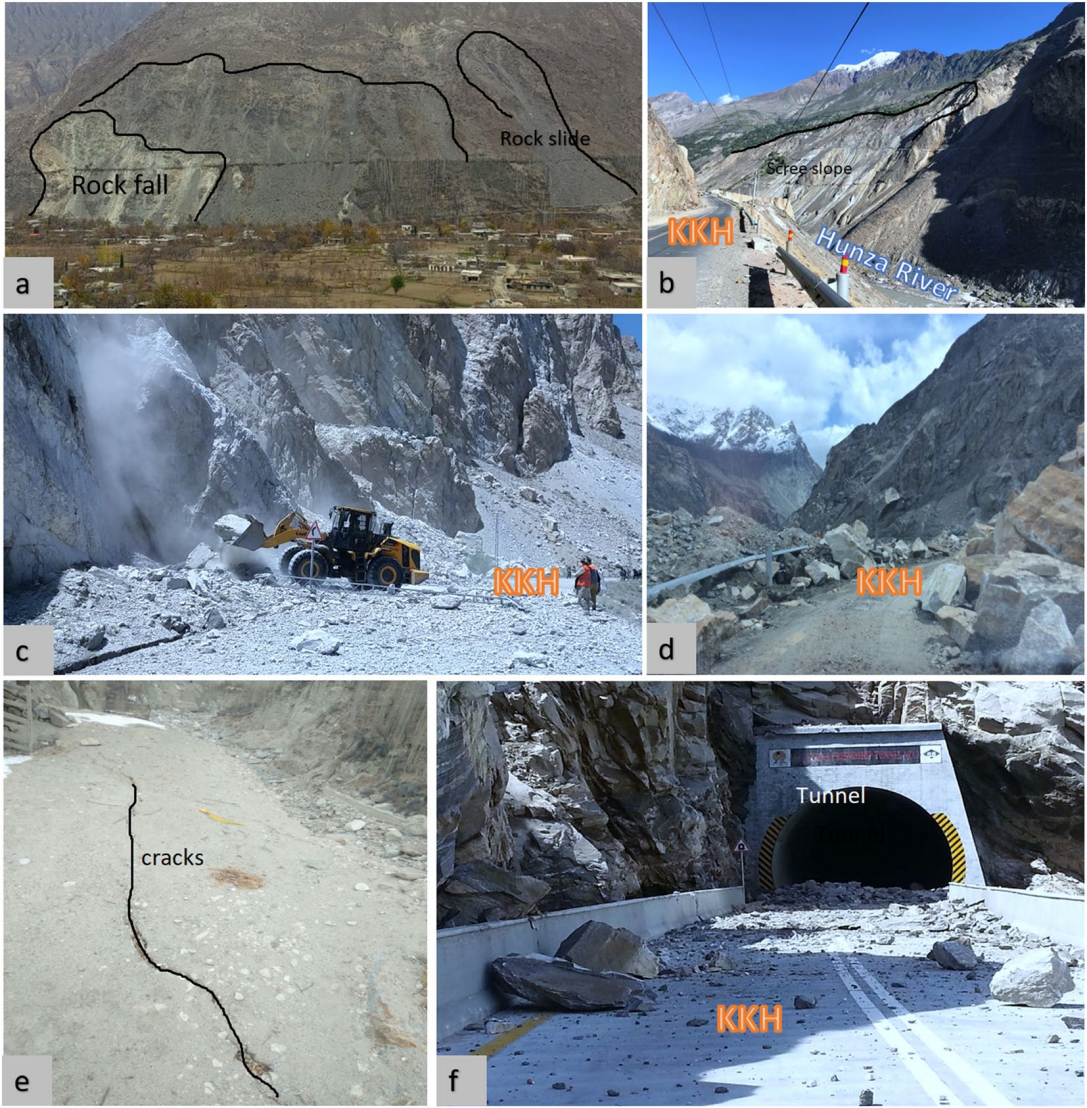
Some recent landslide event along the KKH captured during fieldwork; (**a**) shows a rockfall and rockslide event at Hunza, (**b**) a destabilized slope opposite side of KKH at Nagar-Hunza section, where debris is dumped in the Hunza River, (**c**) a huge landslide blocked KKH at Passu area and (**d**) shows the rainfall-induced landslide at Sost Gojal, (**f**) ground crack captured at Nagar district, and (**e**) Rockfall on the mouth of a tunnel on KKH.

An inventory map has information about the location, date of occurrence, and types of landslides that have left discernible traces in an area^29^. It is a record of all kinds of past landslide events in the area. Landslide inventory maps are developed for a variety of reasons, such as identifying the location and type of landslides in a given area, illustrating the effects of a single landslide-triggering event, such as an earthquake, an intense rainfall event, or a rapid snowmelt event, highlighting the abundance of mass movements, calculating the frequency-area statistics of slope failures, and supplying pertinent data to build landslide susceptibility models or hazard models^30^. In this study, 234 landslides were mapped using past studies^7,8,12,28,31,32^, optical imagery interpretation techniques, and fieldwork of the area (Figs. 4, 5). The inventory map was classified into 8 classes according to the movement of material, which includes 9 complex landslides, 25 debris fall, 91 debris flow, 25 debris slide, 20 rock avalanche, 2 rock fall, 10 rockslide, and 52 scree slope (Fig. 4). This inventory map was applied to validate the InSAR-based detection of landslides in the area. After InSAR processing, some new landslides were mapped according to high deformation velocity (V_slope_) and the inventory map was updated accordingly.

### Landside conditioning parameters

**Figure 6:**
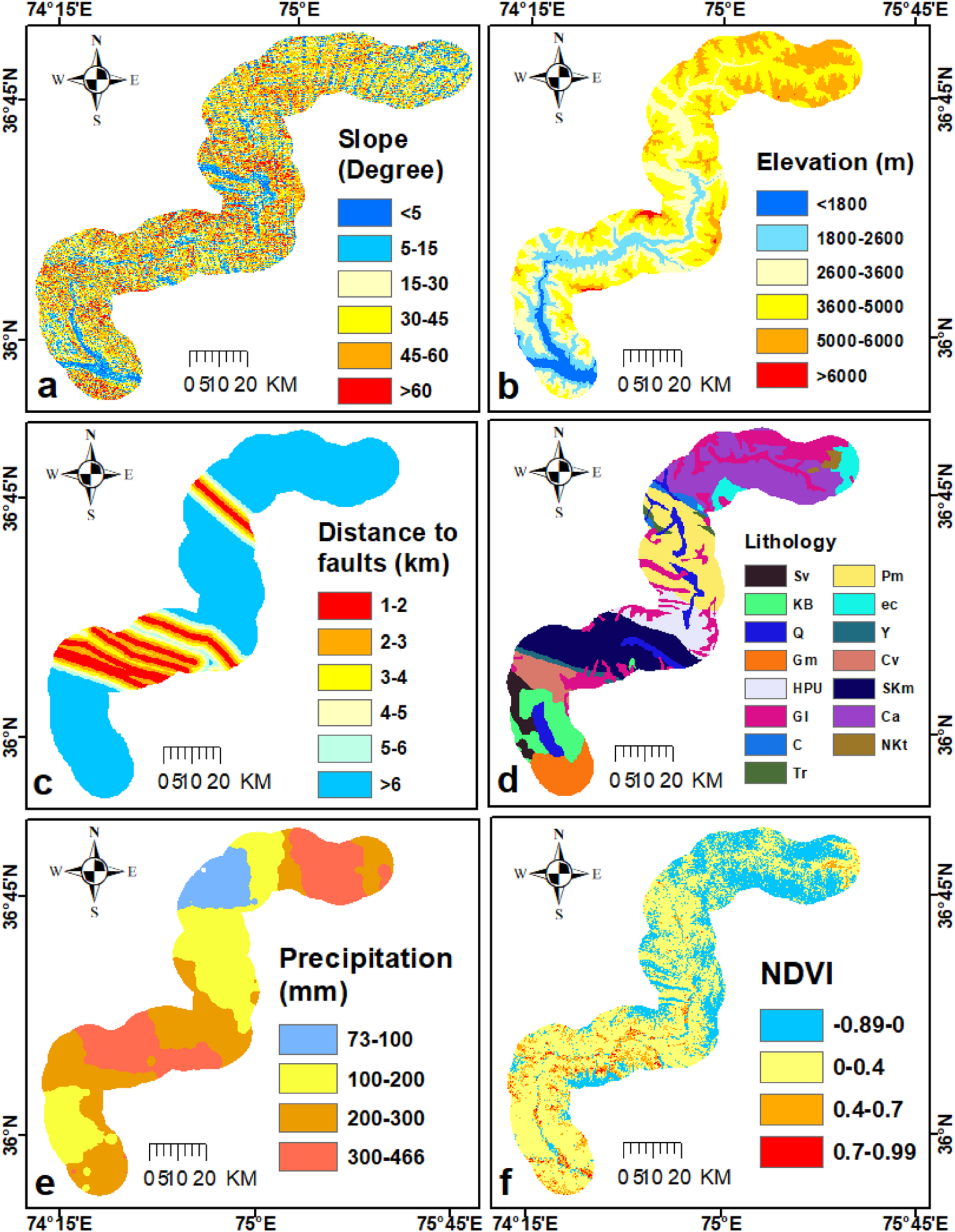
Thematic map of landslide conditioning parameters analyzed in the study shown in four classes with different colors (SRTM DEM 30 m data and Sentinel-2 open accessed data); (**a**) Slope, (**b**) Elevation, (**c**) Distance to fault, (**d**) Lithological units, (**e**) Annual precipitation and (f) NDVI. (©ESA ©USGS).

The terrain, geology, tectonic features, weather, land cover, and anthropogenic variables in the area greatly influence the spatial distribution and intensity of the landslides. Since there is no universal agreement on which triggering parameters should be included in modelling for landslides since they are very difficult-to-understand natural phenomena, it is important to include controlling factors that are pertinent to the study area and have access to accurate data^24^. According to previous studies slope, relief, fault lines, geological setting, precipitation, and barrenness in the area are the most responsible factors in landslide triggering in the study area. In this study, these six factors (Fig. 6) were taken under investigation to check the correlation with the inventory data. The spatial relationship between a landslide occurrence location and each landslide-conditioning factor was derived using the frequency ratio (FR) model. It is crucial to construct a comprehensive landslide inventory consistently over a sufficiently broad spatial extent and a long period of time. Moreover, by providing spatial–temporal coverage, several potential landslide conditioning factors can be statistically associated^33^. The frequency ratio measures the proportion of the study region where landslides happened as well as the probability of a landslide occurring versus not occurring for a particular attribute^34^. FR was calculated using the following equation;

1 $$FR = \frac{dA/dB}{{\sum dA/\sum dB}}$$

where dA is the area of landslides in the given class, dB is the area of the class; ∑dA is the total sum of the area of the landslide in the entire study area, ∑dB is the sum of the entire study area. If the value of FR is greater than 1, it shows a high correlation, and less than 1 means a very low correlation with that class.

### PSInSAR processing

**Figure 7:**
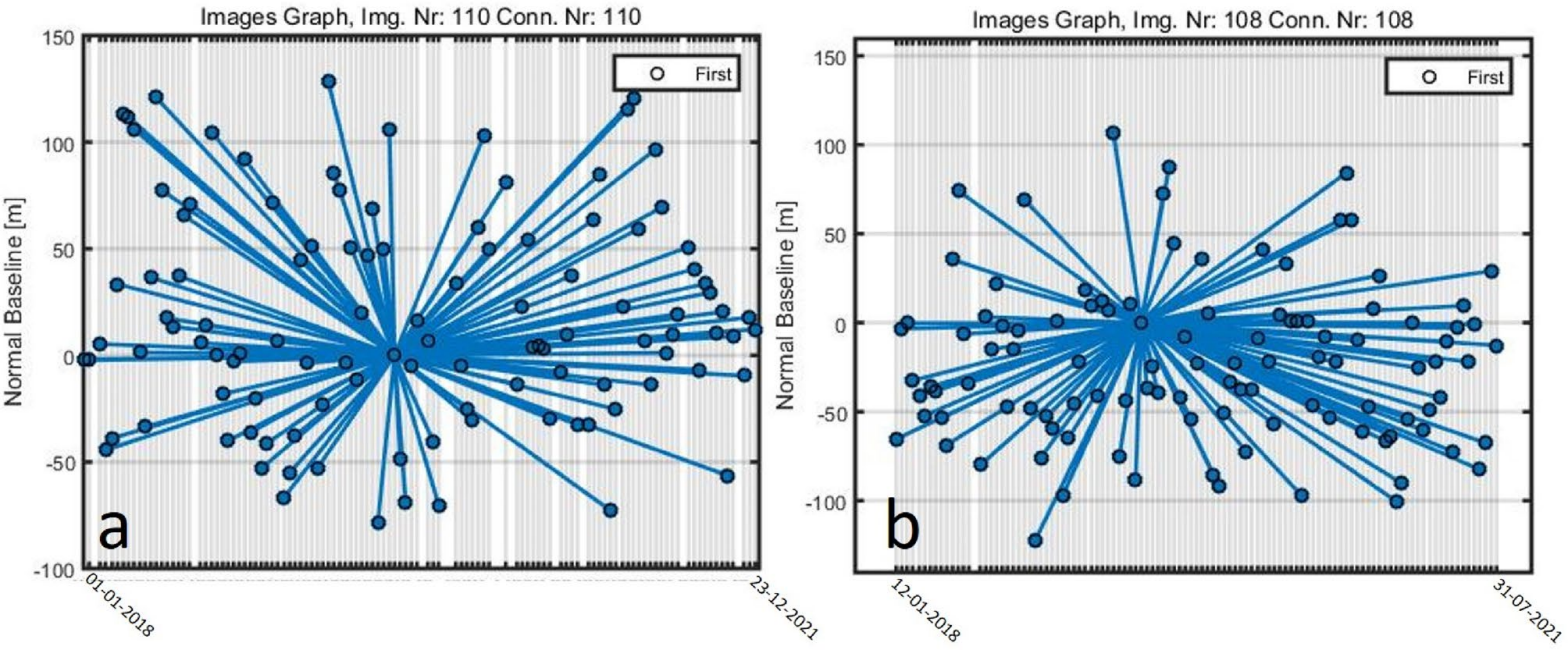
The spatiotemporal baseline of PSInSAR; (**a**) Descending path and (**b**) Ascending path.

The spaceborne interferometric radar technique is a potent instrument for spotting ground motions on the Earth. Due to the excellent cost–benefit ratio, non-invasiveness, large area coverage, and high precision of satellite data analysis, mapping geomorphologic processes and monitoring slope instability can both considerably benefit from it. to detect landslides PSInSAR technique is effective and applied by many researchers in different parts of the world^20–23,35,36^. The Permanent Scatterers (PS) approach uses co-registered, multi-temporal synthetic aperture radar (SAR) imagery (at least 15) to analyse a backscattered signal to find out highly reflecting ground features that are stable from an electromagnetic aspect^20^. The spatiotemporal baseline of PSInSAR have been calculated for descending path and ascending path is shown in Fig. 7. To create two-dimensional complex image maps of the surface with dimensions ranging from slant range to line of sight (LOS) range, SAR systems, which are active, use microwave lights and save the electromagnetic echoes reflected from the surface. The displacement time series and displacement rates of each stable point (PS) can be calculated along the SAR LOS concerning a reference point that is supposed to be stable when a significant number of independent radial light and radial phase stable points (PS) exist within a radar scene and enough radar acquisitions have been collected^37^. The displacements recorded for each PS are calculated using a stable ground point with known coordinates as the reference point. Different sensors having the same wavelength collect data throughout time and can be used in a multi-temporal analysis of ground deformation. This indicates that each PS dataset’s ground deformation velocity is measured along a separate LOS^38^.

PSI focuses on stable-point scatterers (having the same wavelength) that are unaffected by speckles and provide a better signal for analyzing data. Permanent Scatterers (PS) are stable points that return the signal with different durations of time when looking in the same location, which allow for long-term ground subsidence calculation. The interferometric phase (Ø_*Int*_) of a SAR signal of wavelength λ between two different images can be expressed as:

2 $$\emptyset Int = \emptyset topography + \emptyset Movement + \emptyset Noise + \emptyset Atmosphere$$

In the above Eq. (2), Ø_topography_ is represents phase change due to the relief, Ø_*Movement*_ is the terrain motion in the difference between the two different images taken at a different time. Whereas Ø_Noise_ reveals the phase noise which includes other noise components as well and the last is the Ø_Atmosphere_ which represents the phase component due to atmospheric disturbances.

In this research work, Sentinel-1 sensor Single Look Complex (SLC) having polarization VV data was used which consists of a constellation of two polar-orbiting satellites that operate day and night and use C-band synthetic aperture radar imaging to collect data in all weather conditions. A total of 218 images of ascending (108) and descending (110) tracks were processed to measure the velocity along the line of sight (V_LOS_). The imagery acquired on 4 November 2019 was taken as a reference image for descending path processing while the image taken on 24 June 2019 was used as the master image for ascending path. In the step of atmospheric phase screen (APS), 0.75 of amplitude stability index (ASI) threshold was used for the first-order point selection. ASI can be calculated to select PS by applying the following equation;

3 $$ASI = 1 - D_{A} = 1 - \left( {\frac{{\sigma _{A} }}{{m_{A} }}} \right)$$

where D_A_ represents the amplitude dispersion, m_A_ is the mean deviation of amplitude in time, and σ_A_ is the standard deviation of amplitude in time.

These strict criteria are only satisfied by a small subset of points, yet it is essential for accurate APS calculations. This mountainous area utilized only those point targets, which had amplitude dispersion values less than 0.25, the selection of pixels below the 0.25 threshold of ASI allowed selection points of high ASI. This also ensures the selection of only those PS points owning minimum decorrelation noise.

A reference network must be created by linking the PSs using Delaunay triangulation after choosing the first order PS. Each edge's differential residual topographic error (RTE) and differential deformation velocity are calculated. The APS is then computed from the phase residuals by graph inversion after the estimated linear model (linear displacement velocities and residual height) is eliminated. In Second order PS selection, the requirement was lower (ASI > 0.6) to produce a PS collection that was denser, where 213,601 points for descending and 298,827 points for the ascending track were selected. Then, using the same parameters and the same reference point as for APS estimation, the final procedure with APS removal was carried out.

The V_LOS_ was calculated but it does not represent actual ground target displacements. By integrating the geometric relationship between the SAR sensor and the terrain, it is possible to utilize the directions of velocities along the steepest slope gradient (V_slope_) to get over V_LOS_' limits. V_slope_ is thought to represent the direction in which actual deformations caused by potential slope failure will occur most frequently^22^. The rate of deformation in the LOS direction is insufficient for reflecting the true deformation of the slope in mountainous areas^3^. The conversion for V_LOS_ to V_slope_ was done using the below Eq. ^39^.

4 $${V}_{slope}=-\frac{{V}_{LOS}}{Cos\varnothing }$$

where V_LOS_ line of site deformation and Ø is the incident angle of the radar wave.

In the final step, to assess potential landslides deformation velocity was classified. The criteria for threshold selection have been done by different researchers according to the nature of the study area and the purpose of the study by incorporating different InSAR techniques. The degree of activity of landslides can be assessed in order to calculate the average V_slope_ of landslides that contain a significant number of coherence thresholds (CTs) and define the V_slope_ stability threshold statistically^40^. The matrix technique uses multi-temporal InSAR datasets as indicators of the activity and intensity of landslide processes^41^. T0 identify landslide occurrence, a threshold of − 20 mm/y along the slope direction was established^31^ and some other methods have been applied in different studies. Depending on the lithological properties, failure mechanisms, sensor measurement accuracy, and the aims of the inquiry, further interpretation of the observed pattern can lead to the identification of the displacement rate threshold to identify potential landslides and the statistical factors such as the standard deviation of displacement rates are frequently used to inform these criteria^42^. In our study we were aimed to map the high potential landslides, therefore > 25 mm/yr of V_slope_ threshold were implied.

### Landslide susceptibility mapping

The mapping of locations with an equal likelihood of experiencing landslides during a given timeframe is known as landslide susceptibility. The assessment of the terrain's susceptibility for a slope failure, in which the susceptibility of the terrain for a hazardous process expresses the likelihood that such a phenomenon occurs under the specified terrain conditions or parameters, and the estimation of the likelihood of a triggering event constitute a landslide hazard zonation^43^. Analysis of the susceptibility from landslides can help design policies for land use planning and offer helpful information for preventing catastrophic damage. By identifying the factors that affect landslides, estimating the relative contribution of slope failure-causing factors, establishing a relationship between the factors and landslides, and making predictions about future landslide hazards based on that relationship, the analysis is used to understand the factors that affect landslides. For landslide susceptibility mapping, inventory data is most significantly related to mapping of all landslides and spatial accuracy, so InSAR-based updated inventory was used to develop a refined susceptibility map for the area with the incorporation of the FR method in R-studio (Eq. 1).

## Results

### Deformation detection

**Figure 8:**
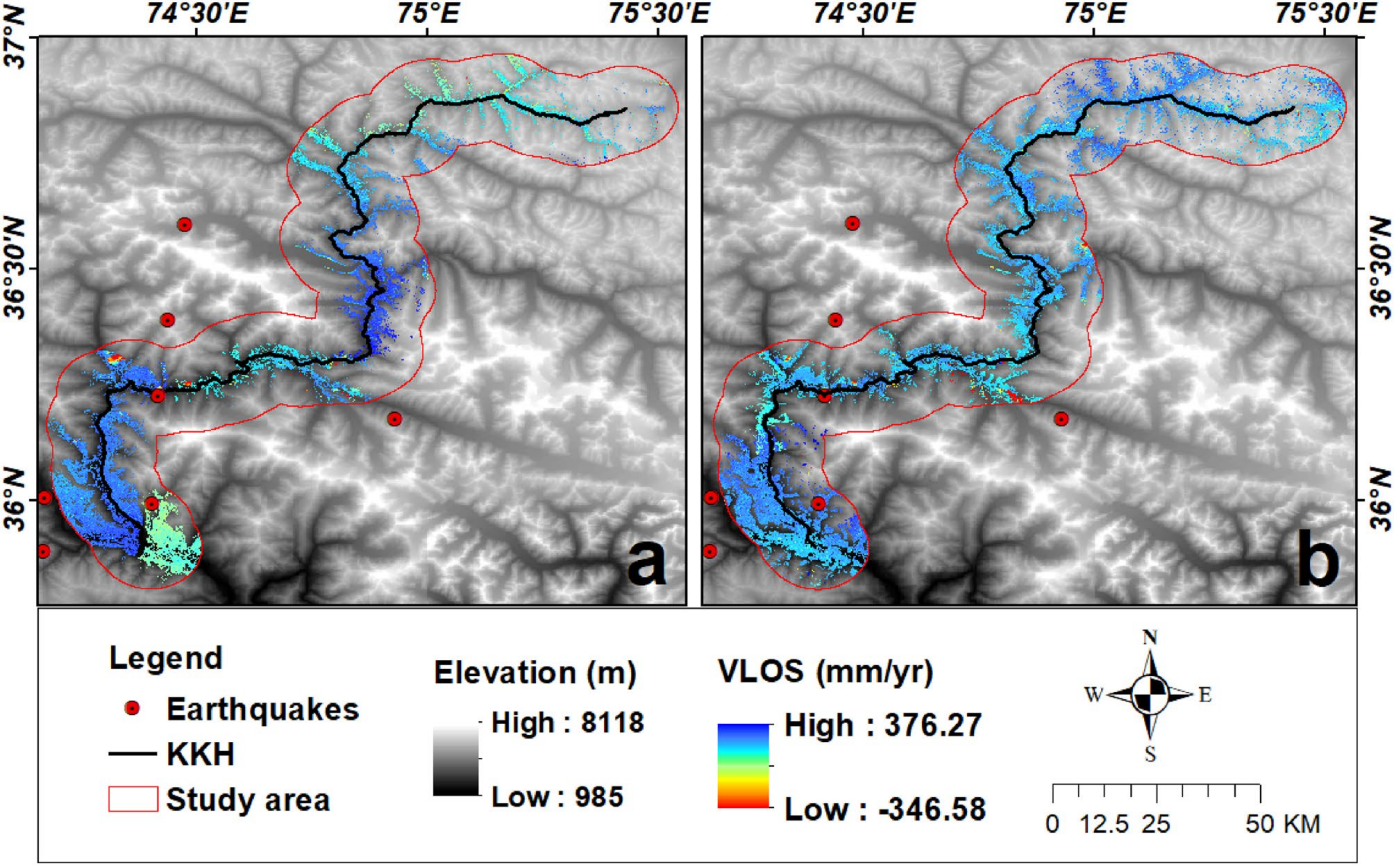
Earth deformation monitoring by PSI approach along the KKH overlaid on DEM; (**a**) is a line of sight movement assessed along the descending track and (**b**) shows a line of sight movement in ascending track. (©USGS).

The Earth's surface deformation from Jan 2018 to Jan 2022 was calculated by using 0.7 as the temporal threshold for coherence. The displacement velocity along the LOS (V_LOS_) is between − 346 and 376 mm/yr for descending track and − 364–364 mm/yr for ascending mode having a total number of 512,428 PS points in the area (Table 1 and Fig. 8). A large number of PS points were found in barren land as compared to vegetated areas.

### Landslide mapped based on deformation velocity (V_slope_)

**Figure 9:**
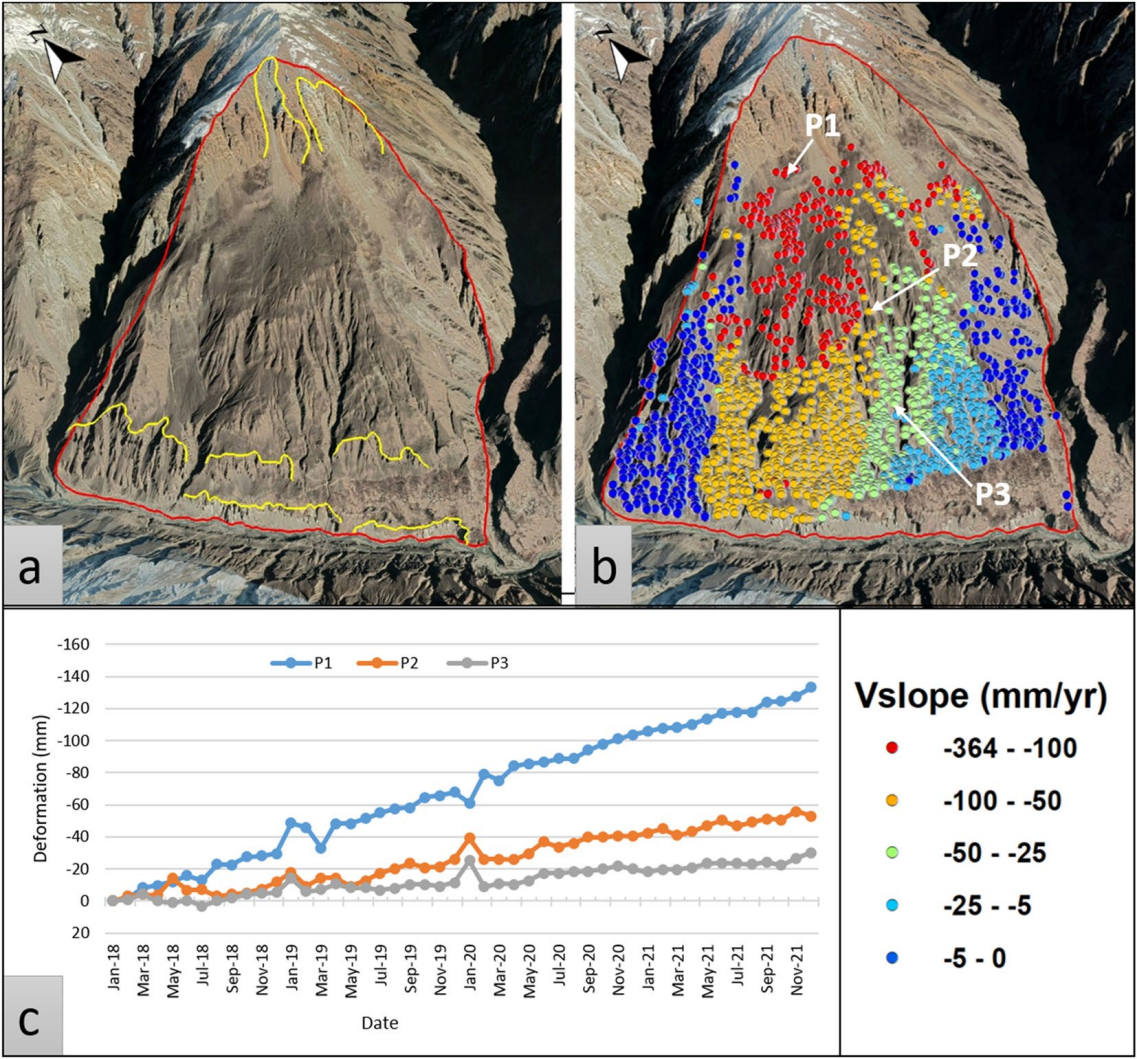
Budalas area complex landslide; (**a**) Optical imagery view, (**b**) Deformation velocity (V_slope_) superimposed on Google Earth imagery for a perspective view, and (**c**) displacement time series for representative PS points p1, p2, and p3.

In this study, the Budalas area was spotted as highly deformed where the mean deformation velocity (Vslope) is > 25 mm/yr (Fig. 9). The displacement movement is towards the south and the slope gradient varies from 15 to 60 degrees. It is a complex landslide, that contains steady velocity from top to bottom (Fig. 9) but the upper part exhibits the highest deformation. The lithology belongs to the Southern Karakoram Metamorphic complex (SKm) which has the most number of landslides in the area^8^, and the area is located near MKT and other small active faults.

**Figure 10:**
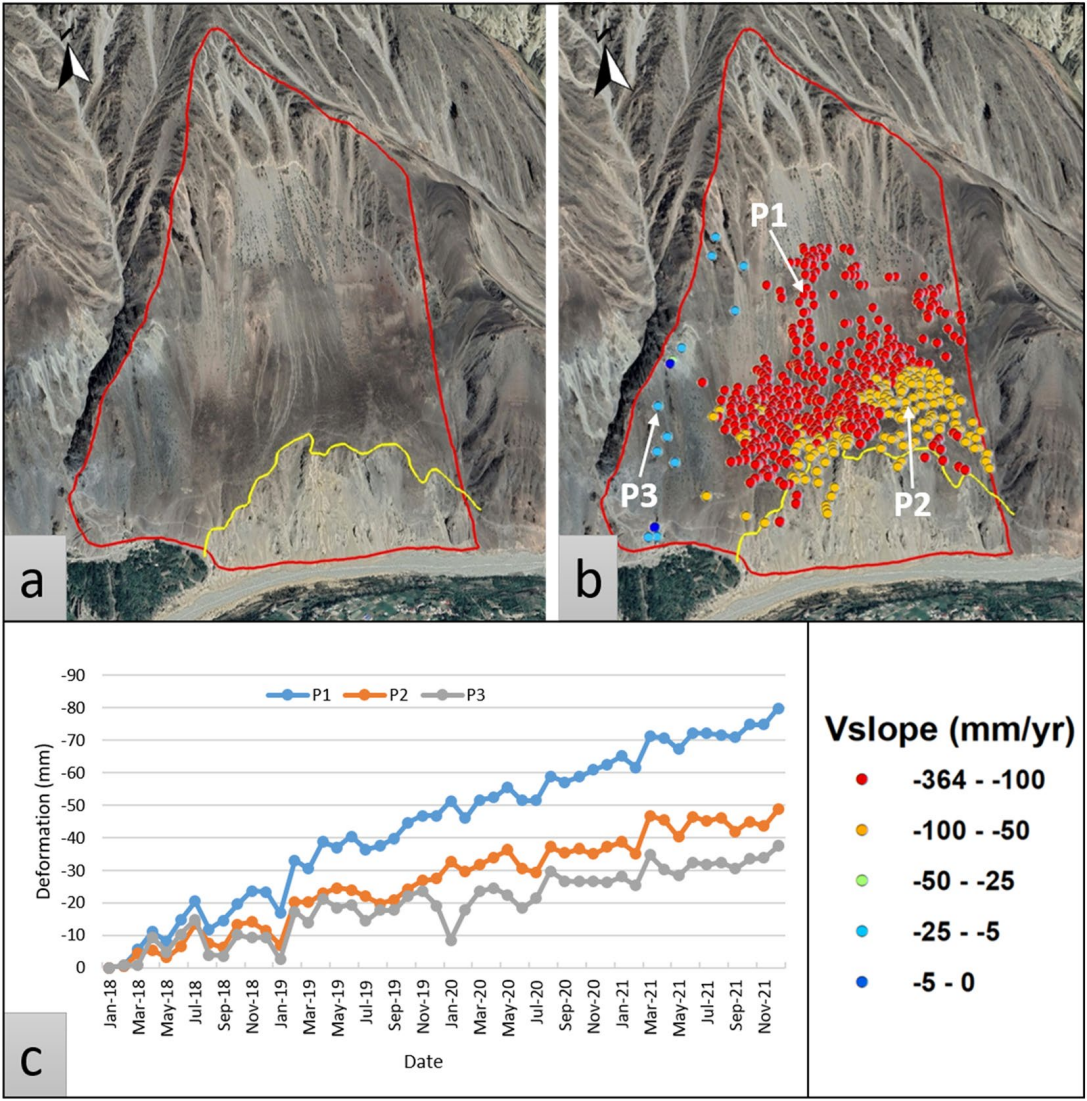
Complex landslide in Khanabad; (**a**) Google Earth view, (**b**) V_slope_ Displacement rate overlaid on optical imagery, and (**c**) displacement time series for PS point p1, p2 and p3.

Khanabad landslide has complex nature and is the most deforming zone in this study possessing a mean Vslope > 50 mm/yr, which was also detected by^12^. The displacement movement is southward and has high deformation velocity at the top. The gradient steepness is from 20 to 65 degrees and is in SKm formation. The lower part has a rockfall slide as seen in optical images (Fig. 10) and the upper side has scree moments.

**Figure 11:**
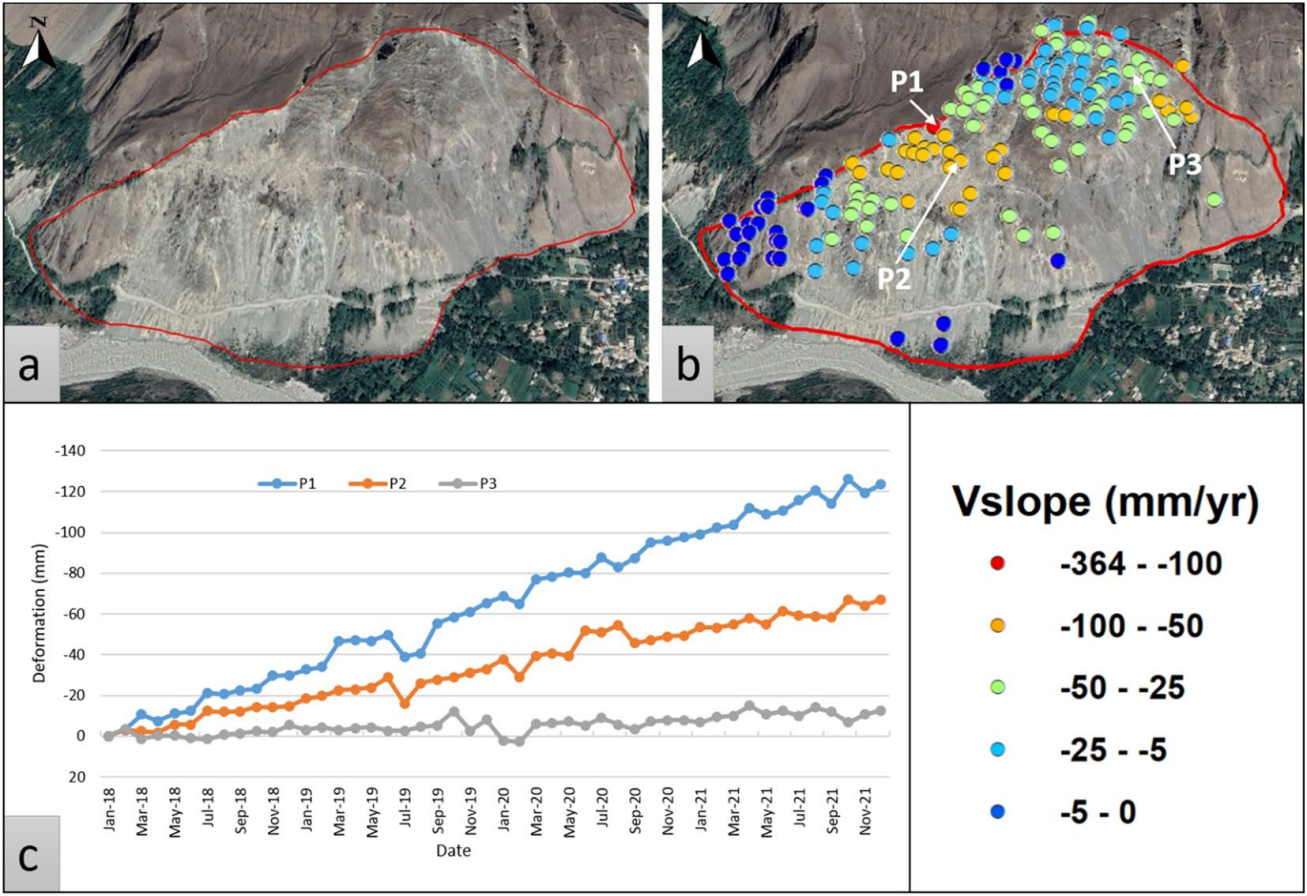
Mayoon landslide; (**a**) Google Earth image of the area, (**b**) slope deformation velocity (V_slope_) superimposed on Google Earth, and (**c**) displacement time series for selected PS points p1, p2, and p3.

Mayoon area has a complex landslide having an average deformation displacement (V_slope_) > 20 mm/yr (Fig. 11). This landslide has several parallel cracks (0.1–5 m opening)^28^ and slides towards the south. The area has 15 to 45 degrees of slope gradient comes in the locality of the Yasin group. In 1976 first landslide was triggered in the area ruining agricultural land to a small extent. The infrastructure was damaged in 2011 when a second slope failure occurred on the eastern end of the escarpment. Twenty families had to be evacuated from the area in 2012 because of a second, comparatively smaller-scale catastrophe^28^.

### Correlation between conditioning parameters and updated inventory

**Figure 12:**
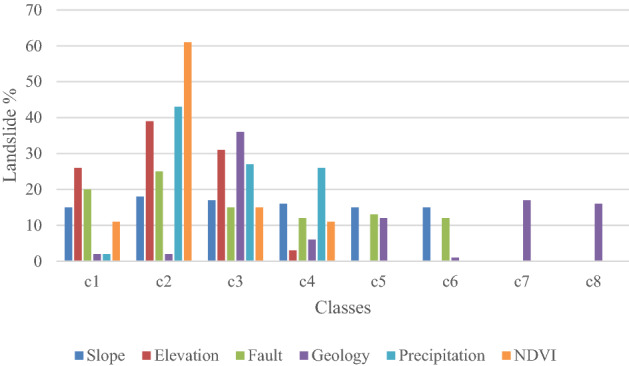
Graphical representation of Table 2. Those classes which possess zero % landslides were ignored as you can see in the classes’ column in Table 2.

The landslide inventory map for the area was converted into a raster form to calculate the number of pixels in different classes of conditioning parameters (Table 2). The assessment showed that, more than 80% of landslides in < 45 degrees of slope (Fig. 12, Table 2). The gradient classes from 15 to 60 degrees are positively correlated with landslide activities because the terraces of the Hunza river accumulated with ancient landslides, which are unstable in the area. According to the statistical estimation the elevation areas from 1500 to 3600 m have around 90% of landslides (Table 2). The class 1800–2600 m and 2600–3600 m have the FR value of 2.56 and 2.05 for landslide, which shows a high correlation. The sloped body having less than 3600 m of relief has seen extensive weathering, and gravity, in addition to fluvial and runoff erosion, is the primary driver of slope collapse. The zones that are higher than 3600 m of elevation are found in exposed bedrock locations that are less impacted by river and rainfall erosion, and hence low numbers of landslides occur.

**Table 2:** Calculated weight for triggering parameters by incorporating updated inventory in Eq. (1).

Parameter	Classes		% pixels in the class(dB)	% Landslide pixels in the class (dA)	FR = dA/dB
Slope (°)	C1	< 5	12.03	11.14	0.93
	C2	5–15	12.52	14.29	1.14
	C3	15–30	16.78	17.81	1.06
	C4	30–45	25.57	26.03	1.02
	C5	45–60	25.31	23.52	0.93
	C6	> 60	7.78	7.22	0.93
Elevation (m)	C1	< 1800	3.95	6.8	1.72
	C2	1800–2600	11.45	29.35	2.56
	C3	2600–3600	26.56	54.52	2.05
	C4	3600–5000	42.61	9.33	0.22
	–	5000–6000	14.99	0	0
	–	> 6000	0.44	0	0
Fault (km)	C1	0–1	10.15	9.48	0.93
	C2	1–2	8.1	9.59	1.18
	C3	2–3	5.24	9.79	1.87
	C4	3–4	4.29	6.4	1.49
	C5	4–5	3.18	3.27	1.03
	C6	> 5	69.04	61.46	0.89
Geology	C1	Sv	2.64	0.6	0.23
	C2	KB	6.68	1.72	0.26
	C3	Q	5.81	23.81	4.1
	C4	Gm	5.57	4.27	0.77
	C5	HPU	5.79	8.25	1.43
	C6	Gl	16.46	3.53	0.21
	–	C	1.02	0	0
	–	Tr	0.69	0	0
	C7	Pm	13.65	26.98	1.98
	–	ec	3.32	0.14	0.04
	–	Y	1.18	0	0
	–	Cv	4.2	0.14	0.03
	C8	SKm	15.53	29.05	1.87
	–	Ca	16.66	1.39	0.08
	–	NKt	0.81	0.09	0.11
Precipitation (mm/yr)	C1	73–100	8.53	0.63	0.07
	C2	100–200	32.15	46.13	1.43
	C3	200–300	34.75	31.48	0.91
	C4	300–466	24.56	21.75	0.89
NDVI	C1	− 0.89	29.65	7.72	0.26
	C2	0–.4	62.62	89.68	1.43
	C3	0.4–0.7	6.73	2.33	0.35
	C4	0.7–0.99	1	0.27	0.27

The area has active tectonic nature and experienced many earthquakes in the past, so on the premises of fault lines several landslides were mapped. Based on the presumption that slopes near a fault are more likely to experience deformation, the distances from various faults were quantitatively estimated, which shows there are many densely populated landslides near active faults (Fig. 12). The strength and deformability of the bedrock are directly impacted by the presence of faults. A distance of less than 5 km is positively correlated with landslides in the area (Table 1). The Quaternary (Q) Deposits, Southern Karakoram metamorphic Complex (SKm) the Hunza Plutonic Unit (HPU), and Permian massive (Pm) limestone have approximately 90% of landslides (Table 2, Fig. 12). In short, deformation hazards mostly occur in sedimentary and metamorphic rock along the KKH.

**Figure 13:**
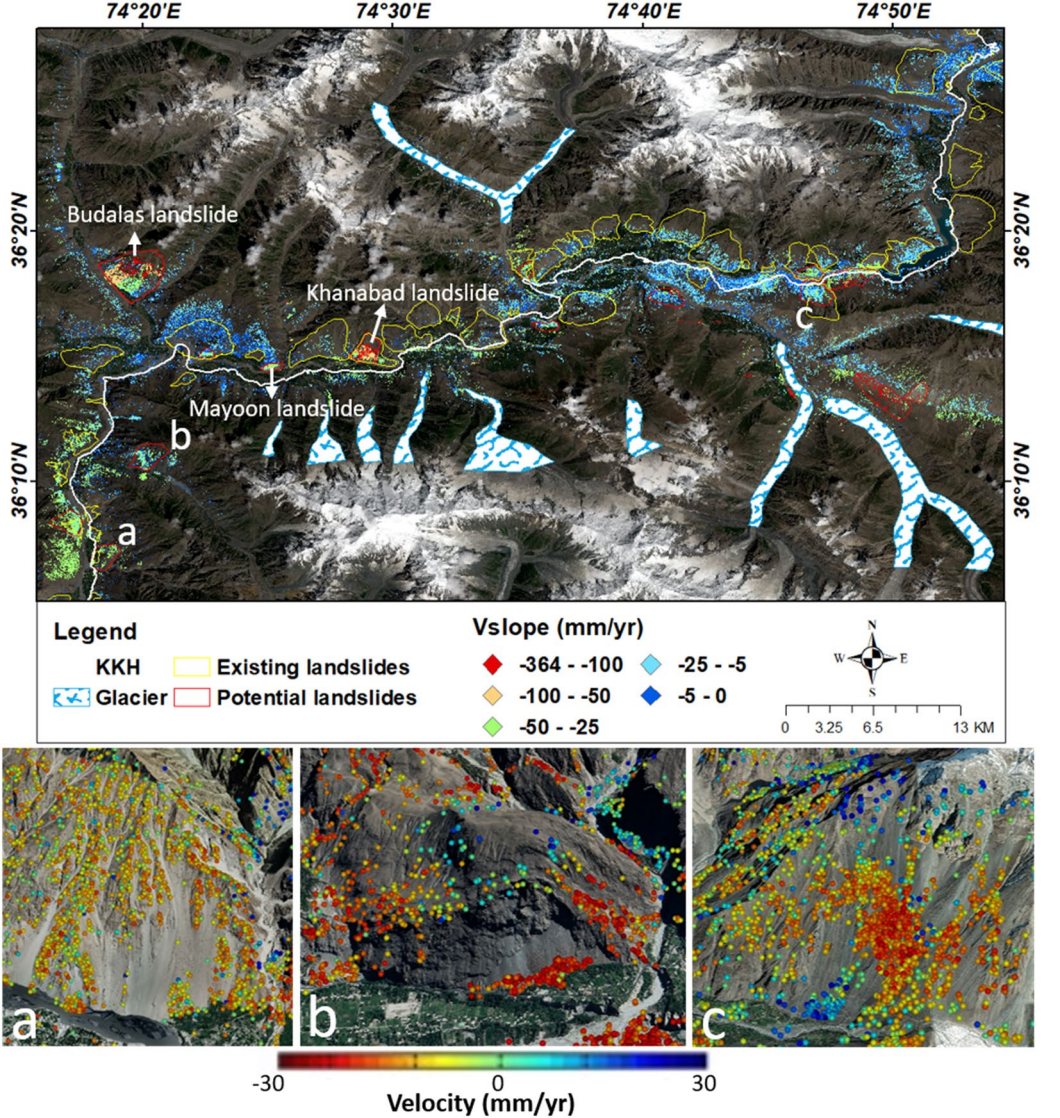
Distribution of potential and documented landslides and glaciers in Hunza valley possess Sentinel-2 imagery as a basemap in ArcGIS environment (**a**, **b** and **c** are the V_LOS_ overlaid view on Google Earth optical imagery of some potential landslides mapped in this study). (©USGS).

**Figure 14:**
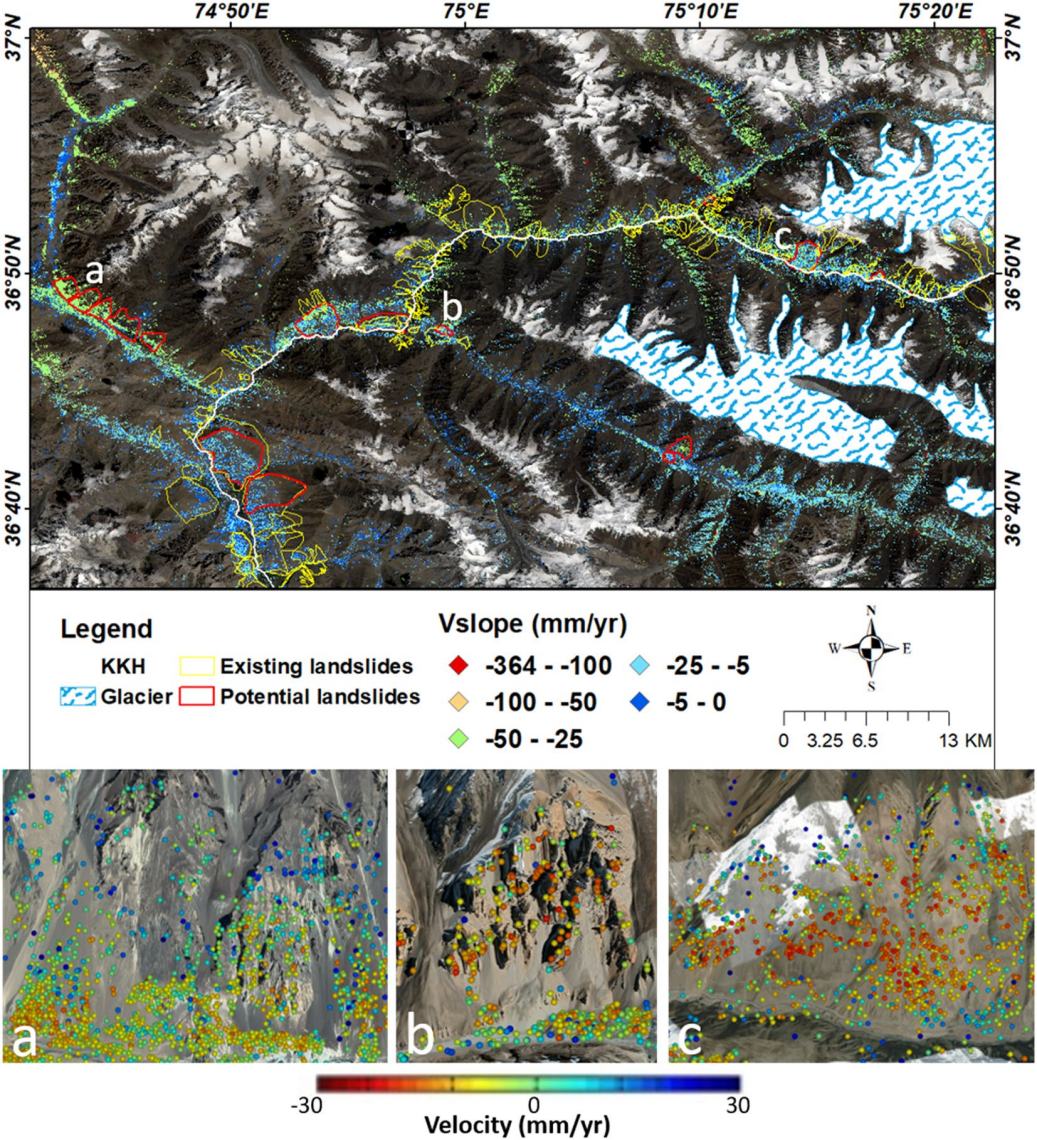
Zoomed view of the distribution of potential and existing landslides in Mighar valley to the upper parts of the study area (a, b and c are the Google earth images of some potential landslides on which V_LOS_ overlaid). (©USGS).

KKH is a semiarid zone have less annual precipitation (Table 2). Statistical analysis shows those areas which experience high rainfall possess a large number of slope failure events (Table 2). There are around 95% of landslides are located in no or sparse vegetated areas (Table 2). The area of KKH is mostly barren, where almost 90 percent of the landslide (existing landslide (Figs. 13, 14) were mapped in non-vegetated zones. PSInSAR method has limitations in vegetation-covered areas, but more than 60 percent of the land is covered with no vegetation, so it can be concluded that vegetation controls the slope stability in the area as also confirmed in previous studies^8,12,28,31^.

### Updated landslide inventory

In this study, most of the existing landslides which were mapped previously have been also detected in InSAR analysis. Some new landslides possessing high deformation velocity (V_slope_) were mapped and several landslides’ boundaries were modified according to PS points and validated during fieldwork. The updated landslide inventory was classified into two classes according to deformation rate. Although most landslides have displacement to some extent (Figs. 13, 14), some zones are deforming at the rate of > 25 mm/yr. Those landslides which have high deformation velocity were included in the potential class and other landslides have low deformation values and have information previously classified into existing landslides (Figs. 13, 14). All these potential deforming slopes are complex having different kinds of downfall movement of material and these sites need further analysis by geoscientists to cope with the Ataabad-like disaster in the future.

### Landslide susceptibility mapping

**Figure 15:**
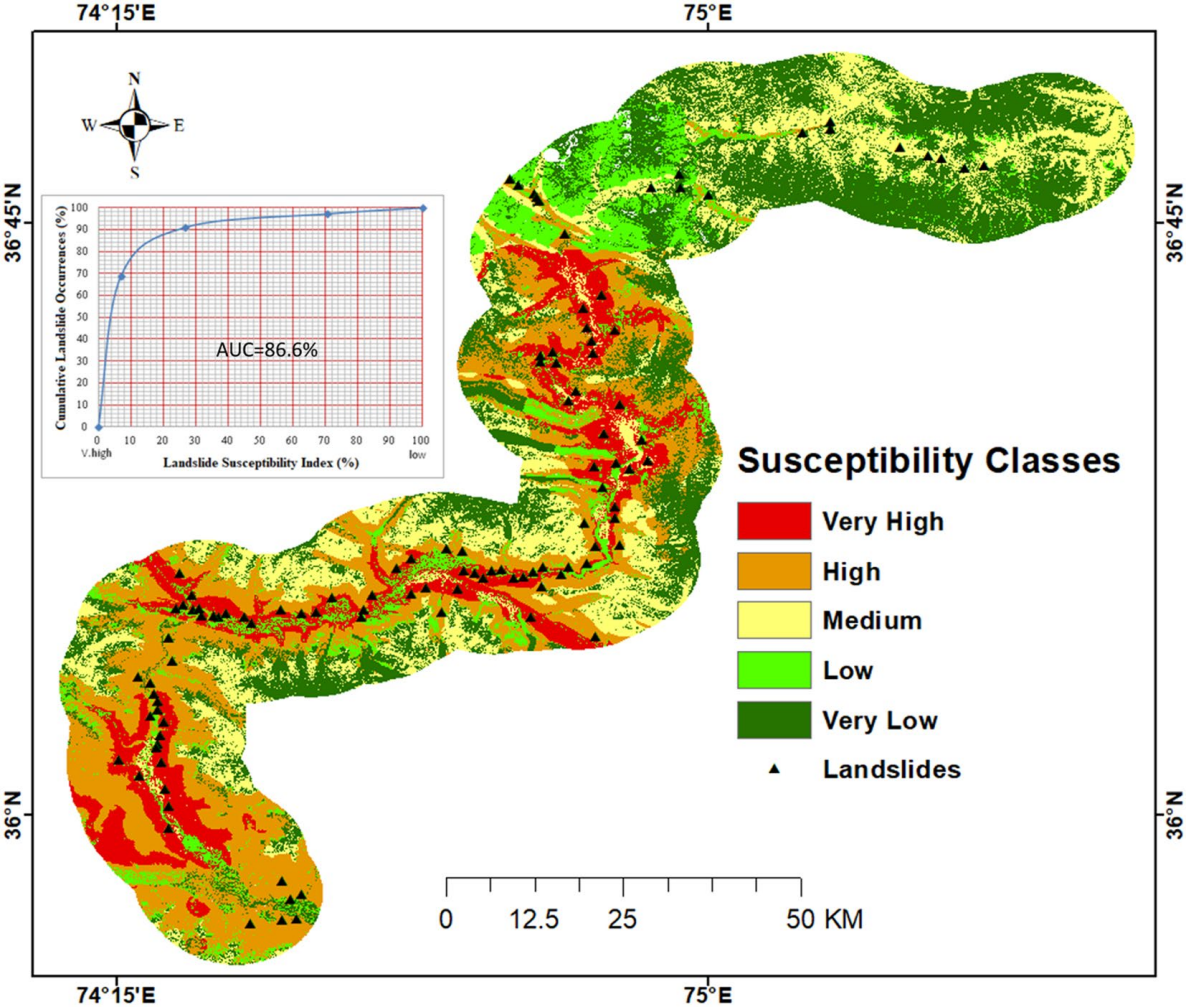
Landslide susceptibility map of the study area classified into five zones from very high to very low with different colors. Black rectangle shows the different locations of landslides. (©USGS).

The updated landslide inventory map and selected conditioning factors were incorporated with the frequency ratio algorithm to generate the susceptibility model for the area (Fig. 15). The accuracy of the model was 86.6 percent calculated by the AUC method (Fig. 15). The susceptibility map portrayed that, Hunza-Nagar and Gojal-Passu areas are the most hazardous for landslide activities. The susceptibility map was classified into five classes from very high to low susceptible zones (Fig. 15).

## Discussion

In this research work, an examination of a Sentinel-1 C-band data set for the potential contribution of PS deformation maps to landslides along the KKH was assessed from Jan. 2018 to Jan 2022. The deformation velocity along the slope (V_slope_) map was incorporated to map a number of new potential landslides and adjustment of limits on documented landslides (Fig. 5). Optical remote sensing data interpretation, existing landslide inventory data, and a fieldwork survey of the area were employed for validation, classification, and further analysis of inventory^12,13,28^.

The previous studies done by applying InSAR were the SBAS method in the area by^12^ using a 1-year temporal period, and^3^ also incorporated the same technique for landslide susceptibility optimization in the China section of KKH. In this study, the PSInSAR approach was tested over 4 years of a temporal period which showed promising results, and the relationship between the mapped landslide body and PSInSAR data reveals a good correlation in the area. In this study, several new landslides were mapped that were missed in previous studies like the Budalas landslide having a mean > 50 mm/yr. These updates offer essential information to end-users and stakeholders for the proper planning of risk mitigation measures because greater PS densities are returned in areas with an urbanized environment and a well-developed road network.

Multi-temporal interferometry has many advantages to studying landslides but also encounter some limitation in different nature of areas. Landslides frequently happen in difficult environmental conditions for temporal InSAR applications (e.g. vegetated slopes, steep and rough topography), where InSAR analysis faces significant uncertainty in estimates of ground motions^44^. This limitation can be solved by the integration of long-wavelength ALOS/PALSAR-2 SAR data for temporal decorrelation in vegetation-covered areas^45^. In this study, it was also assessed that the interferometric processing method is also disabled to detect fast-moving landslides like rockfalls. So, we propose that detailed geohazard monitoring and identification along KKH be accomplished using a variety of approaches and extensive datasets.

Conditioning factors analysis is significant to study landslides. In this research work, the six most responsible factors were evaluated to figure out the correlation with slope failure activities in the area according to all previous studies^7,8,12,32^. In the low terrains, debris flow events are found in many locations along KKH^46^, and 15–60 degrees of sloppy traces were traced to have a more destabilize slope^32^. During rainy seasons loose material from historical landslides flows down and damages KKH frequently. The unstable sections of old landslides in gentle to steep slopes move down slowly found in InSAR results having a huge number of PS points on the historically documented landslides. The barren class was assessed high susceptible to landslides^8^, and more than 60% of the terrains along KKH are barren which weathered with time under the sun, rainfall, and other processes and are prone to landslides. Active tectonic nature accounts for several deformation activities along the fault lines^9^. reported several landslides along the MMT. Several potential deformation events were mapped in the vicinity of the fault lines in this study (Figs. 13, 14), which show faults as a main trigger. KKH is passing through most seismic active part of the world where fault and shear zones show a strong influence on landslide activities in the area^7^.Quaternary Deposits (Q) and Southern Karakoram metamorphic Complex (SKm) lithological units were estimated as more prone to landslide in this work which was also assessed by^8,32^. The area is semiarid but in the monsoon season high precipitation is experienced by the region and debris flow, scree slope failure and sometime rockfall events damage the road every year.

Although there are multiple advantages to using RADAR remote sensing for landslide detection and mapping, but some limitations also exist. Data must be evenly spaced in time (regular sampling). Although InSAR provides a constant revisiting period, it is not always possible for the InSAR time series to provide a constant time interval since it is normal for the loss or omission of some images from processing, which affect the results. The wavelength of the SAR sensor being utilized limits the rate of deformation that can be observed. Catastrophic landslides that are moving quickly may deform at rates considerably above meters per minute. The current generation of SAR satellites cannot directly monitor this extremely fast rate of motion^47^. The abundance of vegetation is another drawback. More vegetative cover causes volumetric decorrelation to rise, which reduces coherence^48^. Coherence could be raised by adding corner reflectors to the steepest or most dangerous vegetated slopes, employing longer wavelengths like L-band that can pass through vegetation, and/or raising the temporal resolution but it also encounters atmospheric delay problems. This could result in inaccurate measures of deformation without the application of sophisticated atmospheric mitigation methods. For this study area, susceptibility maps were forwarded by some researchers previously purely based on quantitative methods, that are dependent on visually interpreted inventory data. In this kind of approach, maximum chances of missing inventory data are possible. To overcome this limitation and to map all kinds of landslides for the development of a complete landslide inventory, optical remote sensing interpretation techniques, fieldwork, and InSAR application for landslide detection were applied and generated a highly accurate landslide susceptibility map for the area.

## Conclusion

This study presents the development of updated landslide inventory and deformation velocity (V_slope_) estimation in a 10 km buffer along the KKH from Gilgit to the Khunjerab section in Northern Pakistan. The results of this investigation show that persistent Scatterer Interferometry (PSInSAR) can significantly update landslide inventories. This InSAR advance works efficiently to modify landslide boundaries, assess their state of activity, and to better understand sliding kinematics. The deformation velocity (V_slope_) was absorbed in 364 mm/yr highest in the area. The Budalas area, Khanabad region, Mayoon landslide, and Attabad area are the highly deforming zones that need to be investigated and mitigate future disasters. Landslides possessing displacement > 25 mm/yr were considered high risk and 29 landslides were mapped and redefined in this study. The PSInSAR-based updated landslide inventory and triggering factor were formulated through the FR model to generate a susceptibility map for the area, which was classified into five zones from very high to low susceptible. Slope, SKm, Q, and Pm lithological units, bareness, and the seismic zones along the fault lines are the most responsible parameters of landslide and deformation activities in the area. The findings of this study can be used for landslide hazard assessment and risk analysis to mitigate the effect and future development planning in the area.

## Data Availability

The data and materials used in this article are available upon request by the correspondence author.

## Abbreviations

SAR, PS-InSAR: Synthetic aperture radar, persistent scatterer interferometric SAR
SRTM: Shuttle radar topography mission
DEM: Digital elevation model
SNR: Signal-to-noise ratio
SPS: Sparse point selection
APS: Atmospheric phase screen
LS: Least square
ScanSAR: Scanning synthetic aperture radar
PSC: Permanent scatter candidates
LOS: Line of sight
mm/year: Millimeter per year
EP: Elevation profile
ESA: European space agency
